# Empowering communities: implementing a COPD self-management program in Nepal

**DOI:** 10.1016/j.lansea.2024.100469

**Published:** 2024-08-16

**Authors:** Saroj Adhikari, Bhuvan Saud, Pravin Kumar Yadav

**Affiliations:** a Government of Nepal, Ministry of Health and Population, Kathmandu, Nepal; b Department of Medical Laboratory Technology, Janamaitri Foundation Institute of Health Sciences, Lalitpur, Nepal; c Physiotherapy Section, National Trauma Center, Kathmandu, Nepal

Globally, Chronic Obstructive Pulmonary Disease (COPD) represents a significant public health and economic burden. In a low-income country like Nepal, COPD prevalence remains the highest in the world, affecting almost 12% of the population.^1^ Additionally, according to the Nepal Burden of Disease Report, COPD accounts for approximately 16% of all deaths due to non-communicable diseases. The age-standardized Disability-Adjusted Life Years (DALY) rate due to COPD is 3318.4, and the mortality rate is 182.5 per 100,000 population.^2^

Acute Exacerbation of COPD (AECOPD) is a significant event among COPD patients that impairs lung function, increases the risk of readmission, decreases the quality of life, results in prolonged hospitalizations, and leads to significant economic burdens and lost productivity. A recent cross-sectional study in a tertiary-level public hospital in Nepal revealed that 68% of COPD patients in the inpatient department had been admitted previously due to AECOPD.^3^ Another observational study highlighted that the length of hospital stay ranged from 6 to 25 days, with high readmission rates further escalating the treatment costs. Nearly all admitted patients (99%) underwent chest X-rays, 88% had arterial blood gas tests, and 76% underwent sputum cultures. Treatment commonly involved short-acting beta-2-agonists (95%), systemic corticosteroids (75%), and antibiotics (97%). Auxiliary treatments included oxygen therapy for all patients, non-invasive ventilation support for over 30% patients and invasive mechanical ventilation for 4% of the patients. About 76% of patients were discharged with long-term oxygen therapy. Moreover, about 37% of patients with AECOPD admitted to hospitals were shown to die within six to seven months post-discharge, and 21% within three months.^4^

The cost associated with the treatment of COPD remains high. Studies across different countries show a wide range of annual COPD costs per capita: from $4398 to $23,049 in Japan, $453 to $12,167 in South Korea, $2700 in Singapore, $4000 in Taiwan, $3942 in China, and $1105 in Thailand.^5^ Additionally, the cost of AECOPD per admission in India is $532.68, with 71% of this being direct hospital costs and 30% related to transportation, medications, diagnostic tests, and out-of-pocket expenses.^6^ Inadequate social health protection in Nepal has resulted in high out-of-pocket health expenditure constituting above 57% of current health expenditure. More than 10% of the population faces catastrophic health expenditure (CHE), and nearly 2% of the population is impoverished due to medical costs. With over 3.4 million people affected by COPD in Nepal, the country must bear substantial economic losses.^7^

The National Health Insurance Program (NHIP) in Nepal offers a benefit package of around $100 to cover medical management and oxygen therapy during admission for COPD exacerbations. Additionally, outpatient visits, diagnostic services and oral medicines are also covered. However, the utilization of health services through NHIP remains low with merely 16% of households enrolled in the program until the fiscal year 2022–23. COPD management is not included in the free basic healthcare package and is available only through the district hospitals. Additionally, the program faces problems pertaining to the availability of services, medicines, equipment, complex referral systems and inadequate readiness of health providers.^8^ As a consequence of these considerable gaps in the social health protection services coupled with the high incidence of multi-dimensional poverty in rural areas (28%) there exists a significant unmet need for COPD patients in Nepal. Around 95% of patients lack access to short-acting beta-agonists (SABA) and 42% lack access to long-acting muscarinic antagonists (LAMA). Additionally, only a small proportion of patients receive guideline-recommended care while many remain undiagnosed.^9^

Several risk factors contribute to the high burden of COPD in Nepal: high prevalence of tobacco smoking, exposure to secondhand smoke, extensive use of biomass fuel for cooking, and poor outdoor air quality.^2^ Meanwhile, the non-pharmacological needs of COPD patients are also critical, with 49% of patients requiring pulmonary rehabilitation, 26% needing reduction of biomass exposure, and 30% requiring smoking cessation support. To address these risk factors and unmet needs, community-level strategy including educational campaigns on the risks of tobacco smoking and biomass fuel along with improving indoor air quality through proper ventilation is crucial. Enhancing health literacy, regular health screenings via spirometry camps and routine check-ups, as well as strengthening primary healthcare services by providing training and necessary resources, are vital.^9^

The social health financing options for Nepal is limited with an annual budget of below 5% of the gross domestic income. In such a situation, health promotion programs like COPD self-management can provide a cost-effective option while contributing significantly to health outcomes. Implementing the program at Primary Health Centers (PHCs), also involving the vast network of Female Community Health Volunteers (FCHVs) can play an important role in educating and supporting the patients and early identification of acute exacerbation. Additionally, a strong referral system between hospitals and PHCs can ensure program uptake, adherence and continuity of care. The key components that enhance the program's efficacy include COPD education, exacerbation action plans, and exercise information.^10^ Patients can enroll at the desired PHC or a basic-level hospital following a back-referral from higher centers once a confirmed diagnosis, appropriate medication and stable condition have been established.

The major program costs include training costs for primary healthcare providers and FCHVs, costs for educational materials including educational mobile application for both providers and patients, and monitoring and evaluation costs. The major benefits include improved quality of life, with other potential benefits: reduced exacerbation rates, reduced hospital admissions and the associated treatment costs. Additionally, the program may contribute to preventing total health expenditure, catastrophic health expenditure and reduce DALYs.^11^

In conclusion, the implementation of a nationwide COPD self-management program at the community level, along with better prevention, diagnosis, treatment, and insurance systems for health expenditures, is essential for addressing the high burden of COPD in Nepal. The cost-effectiveness and potential health outcomes make this health promotion program a valuable choice for Nepal. Additionally, reducing DALYs can contribute significantly to the economy. Moreover, such a program can help Nepal extend social health protection at the community level while achieving several targets of the Sustainable Development Goals (SDGs). This initiative aligns with the strategic action plan of the Nepal Health Sector Strategic Plan 2023–2030 to establish health promotion programs for non-communicable diseases. However, given the socioeconomic context, where many patients are poor and illiterate, and already disabled, the documented benefits observed in high-income countries might not be directly replicable. Therefore, a pilot study should be done to evaluate the actual impact of the program.

## Contributors

Saroj Adhikari conceptualized and prepared the final draft of the manuscript. Bhuvan Saud reviewed and edited the manuscript while Pravin Kumar Yadav reviewed the manuscript.

## Declaration of interests

None.

## References

[1] Karki K.B., Poudyal A., Shrestha N. (2021). Factors associated with chronic obstructive pulmonary diseases in Nepal: evidence from a nationally representative population-based study. Int J Chron Obstruct Pulmon Dis.

[2] Safiri S., Carson-Chahhoud K., Noori M. (2022). Burden of chronic obstructive pulmonary disease and its attributable risk factors in 204 countries and territories, 1990-2019: results from the global burden of disease study 2019. BMJ.

[3] Pokharel P., Lamichhane P., Pant P., Shrestha A.B. (2022). Factors affecting length of hospital stay in chronic obstructive pulmonary disease patients in a tertiary hospital of Nepal: a retrospective cross-sectional study. Ann Med Surg.

[4] Styrvold M., Heggset Sterten A.J., Shrestha S., Dhakal S., Harstad I. (2022). An audit of patients admitted to hospital in Nepal for COPD exacerbation. SAGE Open Med.

[5] Woo L., Smith H.E., Sullivan S.D. (2019). The economic burden of chronic obstructive pulmonary disease in the Asia-Pacific region: a systematic review. Value Health Reg Issues.

[6] Koul P.A., Nowshehri A.A., Khan U.H., Jan R.A., Shah S. (2019). Cost of severe chronic obstructive pulmonary disease exacerbations in a high burden region in North India. Ann Glob health.

[7] NHRC (2022).

[8] Khanal G.N., Bharadwaj B., Upadhyay N., Bhattarai T., Dahal M., Khatri R.B. (2023). Evaluation of the national health insurance program of Nepal: are political promises translated into actions?. Health Res Policy Syst.

[9] Florman K.E., Siddharthan T., Pollard S.L. (2023). Unmet diagnostic and therapeutic opportunities for chronic obstructive pulmonary disease in low-and middle-income countries. Am J Respir Crit Care Med.

[10] Cannon D., Buys N., Sriram K.B., Sharma S., Morris N., Sun J. (2016). The effects of chronic obstructive pulmonary disease self-management interventions on improvement of quality of life in COPD patients: a meta-analysis. Respir Med.

[11] Sánchez-Nieto J.M., Andujar-Espinosa R., Bernabeu-Mora R. (2016). Efficacy of a self-management plan in exacerbations for patients with advanced COPD. Int J Chron Obstruct Pulmon Dis.

